# Methane oxidation linked to chlorite dismutation

**DOI:** 10.3389/fmicb.2014.00275

**Published:** 2014-06-17

**Authors:** Laurence G. Miller, Shaun M. Baesman, Charlotte I. Carlström, John D. Coates, Ronald S. Oremland

**Affiliations:** ^1^United States Geological SurveyMenlo Park, CA, USA; ^2^Department of Plant and Microbial Biology, University of CaliforniaBerkeley, CA, USA

**Keywords:** chlorite, perchlorate, chlorate, methane, oxidation

## Abstract

We examined the potential for CH_4_ oxidation to be coupled with oxygen derived from the dissimilatory reduction of perchlorate, chlorate, or via chlorite (ClO^−^_2_) dismutation. Although dissimilatory reduction of ClO^−^_4_ and ClO^−^_3_ could be inferred from the accumulation of chloride ions either in spent media or in soil slurries prepared from exposed freshwater lake sediment, neither of these oxyanions evoked methane oxidation when added to either anaerobic mixed cultures or soil enriched in methanotrophs. In contrast, ClO^−^_2_ amendment elicited such activity. Methane (0.2 kPa) was completely removed within several days from the headspace of cell suspensions of *Dechloromonas agitata* CKB incubated with either *Methylococcus capsulatus* Bath or *Methylomicrobium album* BG8 in the presence of 5 mM ClO^−^_2_. We also observed complete removal of 0.2 kPa CH_4_ in bottles containing soil enriched in methanotrophs when co-incubated with *D. agitata* CKB and 10 mM ClO^−^_2_. However, to be effective these experiments required physical separation of soil from *D. agitata* CKB to allow for the partitioning of O_2_ liberated from chlorite dismutation into the shared headspace. Although a link between ClO^−^_2_ and CH_4_ consumption was established in soils and cultures, no upstream connection with either ClO^−^_4_ or ClO^−^_3_ was discerned. This result suggests that the release of O_2_ during enzymatic perchlorate reduction was negligible, and that the oxygen produced was unavailable to the aerobic methanotrophs.

## Introduction

*In-situ* production of CO_2_ by microbial activity is encouraged during enhanced oil recovery as a means of reducing oil viscosity and improving flow characteristics (Lazar et al., 2007; Youseff et al., 2009). Further, targeted growth of microbes and intentional precipitation of solid phase minerals can be applied to selectively decrease permeability and direct flow to enhance oil recovery (Jenneman et al., 1984; Zhu et al., 2013). These enhancements rely on the availability of appropriate electron acceptors to supply oxidant to microbes utilizing hydrocarbons or other reduced compounds as electron donors. Considerable attention has been paid thus far to the use of sulfate, nitrate or nitrite as electron acceptors in these applications (Youseff et al., 2009) with sulfate less favored because its reduction results in H_2_S and leads to oil souring (Gieg et al., 2011). There has been recent interest in the use of perchlorate or chlorate, together known as (per)chlorate as electron acceptors. However, little is known about the fate of (per)chlorate in anoxic environments like oil reservoirs. Here we examine the potential reaction of (per)chlorate and chlorite with the low molecular weight hydrocarbon methane.

Chemical reduction of perchlorate is generally quite slow (Urbansky, 2002). However, under anoxic conditions dissimilatory perchlorate reducing bacteria (DPRB) rapidly reduce (per)chlorate to form chlorite. Chlorite thus formed is further degraded by these bacteria using chlorite dismutase to produce Cl^−^ and O_2_ (Rikken et al., 1996; Kostan et al., 2010; Mlynek et al., 2011). These microbial processes reduce (per)chlorate from both natural (Rao et al., 2007; Kounaves et al., 2010) and anthropogenic (Coates and Achenbach, 2004) sources. Degradation of intentionally added (per)chlorate is therefore likely in the proximity of hydrocarbon reservoirs given the abundance of suitable electron donors. There is a potential for O_2_ liberation during this process (see reaction 1 below) that may be used by aerobic bacteria to oxidize aromatic compounds (benzene, naphthalene, catechol) via oxygenase-dependent pathways in otherwise anoxic soils and sediments (Coates et al., 1998, 1999a; Coates and Achenbach, 2004; Weelink et al., 2007; Carlström et al., 2013). A similar phenomenon was noted that could link biological oxidation of arsenite to the reduction of chlorate ions, presumably also by liberation of O_2_ (Sun et al., 2010). This type of interaction has not been extended to the oxidation of low molecular weight hydrocarbons such as methane (CH_4_).

Methane is produced by geothermal and microbial processes in the Earth's crust (Martini et al., 1996) and in marine and terrestrial sediments (Cicerone and Oremland, 1988). Methane is removed photochemically in the atmosphere by reaction with hydroxyl radicals but the most important removal mechanism in aqueous and terrestrial environments is by the action of anaerobic and aerobic methane oxidizing microbes (Cicerone and Oremland, 1988; Boetius et al., 2000). Significant quantities of methane are associated with oil reservoirs (Jones et al., 2007; Gieg et al., 2008) hence we hypothesize that ClO^−^_2_ disproportionation, and by extension dissimilatory reduction of the upstream ClO^−^_4_ or ClO^−^_3_ ions, could be linked to aerobic CH_4_ oxidation by a biochemical release of O_2_:

(1) $${\text{ClO}}_{2}^{-}\;\to \;{\text{Cl}}^{-}+{\text{O}}_{2}\;\Delta \text{G}{{}^{\circ}}^{\prime }\;=-135\text{ kJ/mol }{\text{ClO}}_{2}^{-}$$

(2) $$\underline{{\text{CH}}_{4}+2{\text{O}}_{2}\;\to \;{\text{CO}}_{2}+2{\text{H}}_{2}\text{O }\Delta \text{G }{{}^{\circ}}^{\prime }\;=\;-842\text{ kJ/mol }{\text{CH}}_{4}\;\;\;\;\;\;\;\;}$$

(3) $$\begin{array}{l} \;\text{Net }{\text{CH}}_{4}\text{​}+\text{​}2{\text{ClO}}_{2}^{-}\to {\text{CO}}_{2}+2{\text{Cl}}^{-}+2{\text{H}}_{2}\text{O}\\ \;\Delta \text{G}{{}^{\circ}}^{\prime }=\;-1114\text{kJ/mol }{\text{CH}}_{4} \end{array}$$

Two other well-studied microbiological processes can achieve a net oxidation of CH_4_ under prevailing anaerobic conditions, (1) a reverse process of methanogenesis involving “ANME” archaea in syntrophy with bacterial sulfate- or sulfur-reduction (Hinrichs et al., 1999; Boetius et al., 2000; Milucka et al., 2012) and (2) nitrite-linked CH_4_ oxidation that putatively liberates O_2_ via NO dismutation as achieved by *Methylomirabilis oxyfera* (Ettwig et al., 2010). In our study we explored the potential for aerobic CH_4_ oxidizing bacteria to utilize oxygen produced by DPRB during (per)chlorate reduction and chlorite dismutation.

## Materials and methods

### Preparation of cultures

The DPRB *Dechloromonas agitata* CKB was grown at 30°C under N_2_ on 20 mM sodium acetate and 10 mM NaClO_4_ using phosphate buffer media (PBM) consisting of the following salts in solution (g/liter): Na_2_HPO_4_ (0.971), NaH_2_PO_4_ (0.379), NH_4_Cl (0.25) plus 10 ml/l vitamins and 10 ml/l mineral stock solution (Sun et al., 2009). *Methylococcus capsulatus* Bath, *Methylosinus trichosporium* OB3b, and *Methylomicrobium album* BG8, were grown and maintained at 30°C on air + 30 kPa CH_4_ using nitrate mineral salts media (NMS; Whittenbury et al., 1970). Cultures (1 l) for washed cell suspensions were harvested during late exponential phase, centrifuged (7000 × g), and washed twice with medium lacking substrates and vitamins. Final suspension volumes ranged from 5 to 150 ml. Cell concentrations at the start of incubations ranged from 1.8 × 10^8^ cells ml^−1^ to 6.9 × 10^8^ cells ml^−1^.

### Preparation of soils and slurries

Soil from the seasonally exposed shoreline of Searsville Lake previously shown to harbor methanotrophic activity (Oremland and Culbertson, 1992) was air dried for two days at room temperature before sieving (<1 mm) to assure uniformity of soil particle size. Dried soil was stored for several weeks in stoppered 1 l glass flasks with air headspace and periodically augmented with 0.2 kPa CH_4_ after consumption had removed all of the previously added CH_4_ (4–6 days). Soil with thusly enhanced methanotrophic activity was used to determine CH_4_ uptake in studies with added ClO^−^_4_ and ClO^−^_2_ in the absence of O_2_.

Sediment slurries were prepared by adding 100 ml SeFr2 freshwater media (flushed with 20 kPa CO_2_/80 kPa N_2_; Miller et al., 2013) to 10 g Searsville Lake soil in N_2_ flushed serum bottles (160 ml). Slurry pH was adjusted to 7.1 using 1 ml of 1 M NaHCO_3_. Slurries were incubated under N_2_ headspace following periodic amendments with 1 to 2 mmoles acetate and 0.5 to 1 mmole ClO^−^_4_. Slurries were periodically sampled by syringe using 22 g needles and filtered through a 0.2 um Spin-X centrifuge tube. Acetate and ClO^−^_4_ amendments were made after both were depleted (usually several days to weeks) during which time copious quantities of CH_4_ were produced. Slurries with enhanced perchlorate reducing activity were used to determine methane uptake activity in the presence or absence of added O_2_.

### Measurement of (per)chlorate reduction and chlorite dismutase activity

Aliquots of washed cell suspension of *D. agitata* CKB were distributed into stoppered and N_2_ flushed 25 ml Balch tubes containing 10 ml PBM amended with 5 mM acetate and 10 mM NaClO_4_, NaClO_3_, or NaClO_2_. Initial cell densities were 1.8 × 10^8^ cells per ml. The headspace was sampled over 7 days by syringe for CO_2_ and O_2_. Aqueous samples (0.3 ml) were collected by syringe for analysis of dissolved acetate and anions (Cl^−^, ClO^−^_4_, ClO^−^_3_, and ClO^−^_2_). A short-term (10 min) experiment was conducted to follow ClO^−^_2_ disproportionation. In this study, triplicate samples were sacrificed at pre-determined times. Activity was stopped by addition of 0.1 ml 4N NaOH before measurement of headspace O_2_. Aqueous samples were subsequently collected for analysis of dissolved anions (Cl^−^ and ClO^−^_2_).

### Incubations with mixed cultures

Mixtures (10 ml) of *M. capsulatus* Bath and *D. agitata* CKB were prepared by adding washed cell suspensions of the cultures together in N_2_ flushed Balch tubes (25 ml) sealed with butyl rubber stoppers. Methane (0.2 kPa) was introduced by syringe to all tubes and NaClO_2_ (5 mM) was added to 3 tubes at the start of the incubation which was conducted at 37°C. Headspace CH_4_ was monitored over 1 day. Single tubes were prepared without addition of ClO^−^_2_ or without one of the cultures (i.e., no *D. agitata* CKB or no *M. capsulatus* Bath) to act as negative controls. A tube containing only *M. capsulatus* Bath under an air headspace acted as a positive control.

Additional microcosms were prepared in serum bottles (37 or 67 ml) using washed cell suspensions of *D. agitata* CKB and *M. trichosporium* OB3b or *M. album* BG8. Inocula were either combined in bottles (5 ml each) in one aqueous phase or kept separate by placing methanotrophs (1 ml) inside an open-topped glass tube contained within the bottles before flushing with N_2_ and later adding *D. agitata* CKB (5 ml) by syringe to the bottom of the bottles. In this manner, the cultures were segregated but shared a common headspace. Methane (0.2 kPa) was introduced by syringe to all bottles and NaClO_4_, NaClO_3_, or NaClO_2_ (5 or 10 mM) was added aseptically to start the incubations which were conducted at 28°C. Headspace CH_4_ and aqueous anions were monitored over time. Controls were prepared without additions of ClO^−^_2_.

### Incubations with ^14^C-labeled CH_4_

Washed cell suspensions (5 ml each) of *D. agitata* CKB and *M. trichosporium* OB3b were added together to N_2_ flushed serum bottles (13 ml). Radiolabeled ^14^CH_4_ (5 μCi; specific activity = 21 μCi/μmole) was added along with 1 kPa CH_4_ to the headspace of each bottle. Perchlorate (5 mM) was added to triplicate bottles and ClO^−^_2_ (5 mM) was added to a single bottle by syringe to start the incubation which was conducted at 30°C. Gas samples for analysis of ^14^CH_4_ and ^14^CO_2_ were collected by syringe. At the end of the incubation, samples were acidified using 0.5 ml of 1.2N HCl to cause dissolved inorganic carbon (DIC = HCO^−^_3_ + CO^−2^_3_) to react to form CO_2_ gas which was partitioned into the headspace. The headspace was again sampled by syringe. Control incubations consisted of single bottles of *M. trichosporium* OB3b alone under an air headspace and *D. agitata* CKB alone under N_2_.

### Incubations with soil slurries

Slurry microcosms were prepared in N_2_ flushed serum bottles (57 ml) containing 5 g of dried Searsville Lake soil with enhanced methanotrophic activity to which 10 ml Searsville Lake sediment slurry with enhanced perchlorate reducing activity (above) was added. Half the bottles were maintained under N_2_ while half were flushed with air. Substrate (5 mM ClO^−^_4_ or ClO^−^_2_) was added by syringe followed by 0.5 ml CH_4_ (1 kPa). Incubations were conducted at 22°C. Headspace and liquid samples were collected by syringe over 9 days.

### Incubations with cultures plus soil

Soil microcosms were prepared in serum bottles (67 ml) using washed cell suspensions of *D. agitata* CKB and dried Searsville Lake soil which was enhanced in methanotrophic activity (above). Soil (2 g) was placed inside open-topped glass tubes contained within the bottles prior to sealing and flushing with N_2_. Subsequently, *D. agitata* CKB (10 ml) was added by syringe to the bottom of the bottles followed by aseptic addition of 5 mM acetate. The culture and the soil were thus segregated under a common headspace. Methane (0.1 kPa) was introduced by syringe to all bottles and 10 mM NaClO4, NaClO_3_, or NaClO^−^_2_ was added to *D. agitata* CKB to start the incubations. Incubations were conducted at 22°C. Headspace CH_4_ and CO_2_ and aqueous acetate and anions were monitored over 7 days.

### Analytical

Headspace O_2_ was determined by ECD-GC using a molecular sieve 5A column (3.2 mm O.D. × 2.4 m) operated at 75°C using hydrocarbon-free UHP N_2_ carrier. Background O_2_ was minimized by flushing syringes and needles with O_2_-free N_2_ prior to sampling. The detection limit was 0.05 mmol O_2_/L. Headspace CH_4_ and CO_2_ were determined by FID- and TCD-GC, respectively (Miller et al., 2013). Cell densities were determined by direct cell counting of liquid samples using acridine orange epi-fluorescence microscopy (Hobbie et al., 1977). Additional aqueous samples, including slurries, were filtered using Spin-X centrifuge filter tubes (0.2 μm; Corning Inc., Corning, NY) before determination of dissolved acetate by HPLC (Hoeft et al., 2004) or anions by IC (Miller et al., 2003). Dissolved ClO^−^_4_ was analyzed separately by suppressed conductivity IC using a Dionex ISC 1100 containing an AS16 analytical column (4 × 250 mm) and an AG16 guard column (4 × 50 mm) with 0.035 M NaOH eluent. Measurements of headspace ^14^CH_4_ and ^14^CO_2_ were made by gas proportional counting (Culbertson et al., 1981) following TCD-GC analysis of CH_4_ and CO_2_ with separation on a Hayesep D column (100/120; 3.2 mm O.D. × 4.8 m) using UHP He carrier.

### Calculations

The total amount of gas in each bottle or tube was calculated from the headspace concentration using Henry's Law and the volumes of gas and liquid present. The dimensionless Henry's Law constants (K_*H*_ = C_*G*_/C_*L*_) used were 31.43 for O_2_, 29.46 for CH_4_ and 1.20 for CO_2_ and were not corrected for ionic strength.

## Results

### (Per)chlorate reductase and chlorite dismutase activity

**Figure 1:**
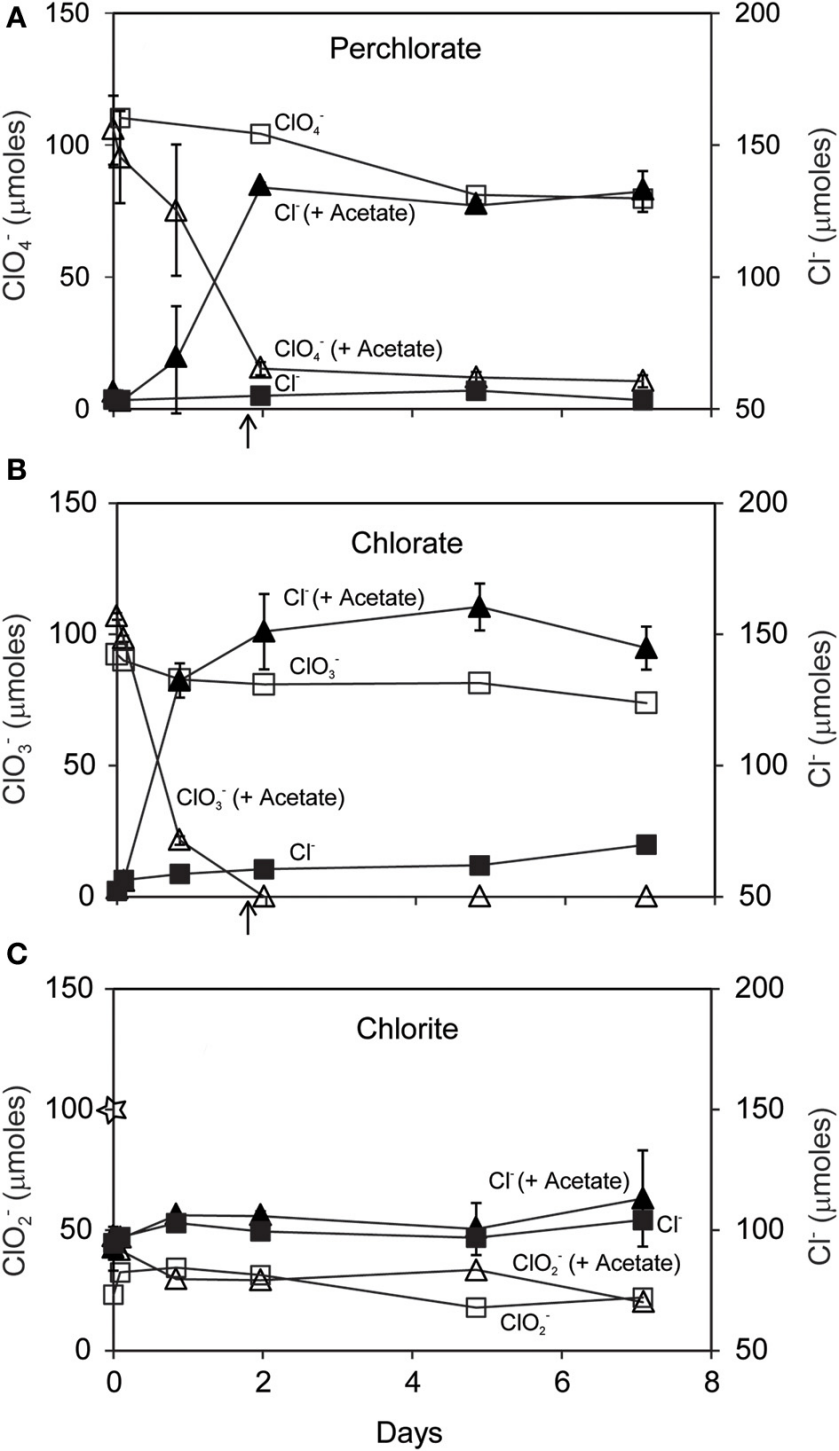
**Time course of reaction of *D. agitata* CKB following addition of 10 mM ClO^−^_4_ (A), ClO^−^_3_ (B), or ClO^−^_2_ (C) showing consumption of added substrate (open symbols) and production of Cl^−^ (closed symbols)**. Triangles symbolize incubations with added acetate (5 mM) while squares symbolize incubations without added acetate. Triangles represent the mean and range of duplicate samples. Absence of bars indicates that the error is smaller than the symbol size. Squares represent single samples. Arrows in **(A)** and **(B)** correspond to the time when 5 mM added acetate was completely consumed. The star in **(C)** corresponds to the initial amount of ClO^−^_2_ added.

Dissimilatory (per)chlorate reduction by *D. agitata* CKB resulted in conversion of 85–100 μmoles added ClO^−^_4_ or ClO^−^_3_ to Cl^−^ in the presence of 50 μmoles added acetate (Figures 1A,B). Much less ClO^−^_4_ or ClO^−^_3_ (<15 μmoles) was consumed without added acetate and a corresponding lesser amount of Cl^−^ was produced. These observations suggest endogenous metabolism of intrinsic electron donors such as glycogen or polyhydroxybutyrate (PHB). No activity was observed in killed controls or in incubations with media and chloroxyanions alone (data not shown). Biological reduction of ClO^−^_4_ or ClO^−^_3_ and consumption of acetate occurred over approximately 2 days. Chlorite dismutation was much more rapid. More than half of the 100 μmoles ClO^−^_2_ added was consumed and converted to Cl^−^ before the initial sampling at *T* = 2 min (Figure 1C). An additional 20 μmoles ClO^−^_2_ were consumed over 7 days, however more than 20 μmoles ClO^−^_2_ remained unreacted at the end.

**Figure 2:**
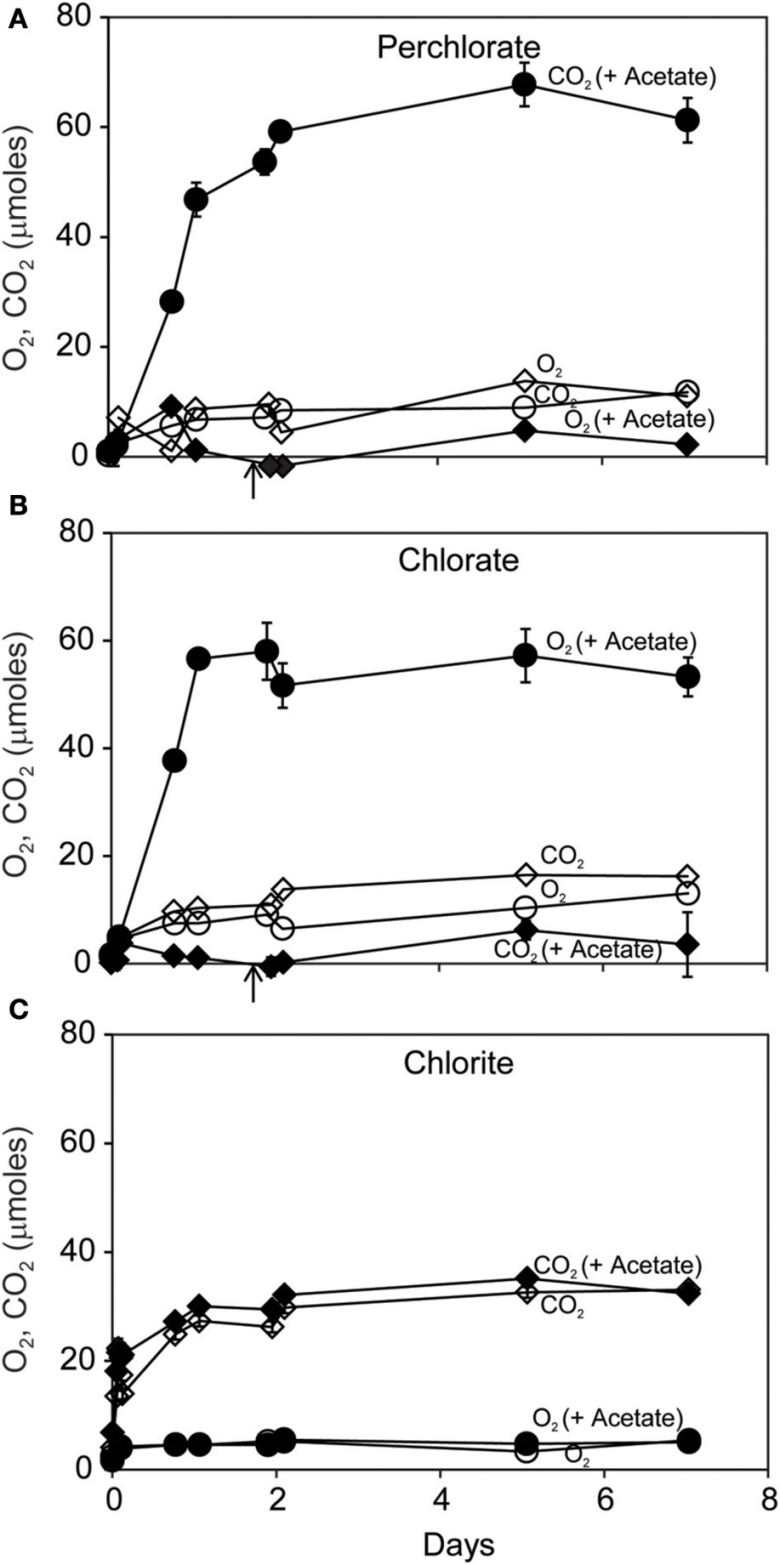
**Time course of reaction of *D. agitata* CKB following addition of 10 mM ClO^−^_4_ (A), ClO^−^_3_ (B), or ClO^−^_2_ (C) showing production of gaseous products O_2_ (diamonds) and CO_2_ (circles), with (solid symbols), and without (open symbols) 5 mM acetate**. Solid symbols represent the mean and range of duplicate samples. Absence of bars indicates that the error is smaller than the symbol size. Open symbols represent single samples. Arrows in **(A)** and **(B)** correspond to the time when 5 mM added acetate was completely consumed.

Carbon dioxide (CO_2_) was the dominant gaseous product of dissimilatory reduction of ClO^−^_4_ or ClO^−^_3_ in the presence of acetate (Figures 2A,B). Slightly more CO_2_ was produced than could be accounted for by the added acetate. Little CO_2_ was produced without added acetate. Small amounts of O_2_ (up to 15 μmoles) were produced during incubations with ClO^−^_4_ or ClO^−^_3_. In contrast, ClO^−^_2_ dismutation resulted in substantial and rapid O_2_ production (Figure 2C) corresponding to release of >35% of the added ClO^−^_2_ within the first day. As expected, there was no effect of added acetate on disproportionation of ClO^−^_2_; however details of the early evolution of O_2_ were obscured by the coarse sampling schedule.

**Figure 3:**
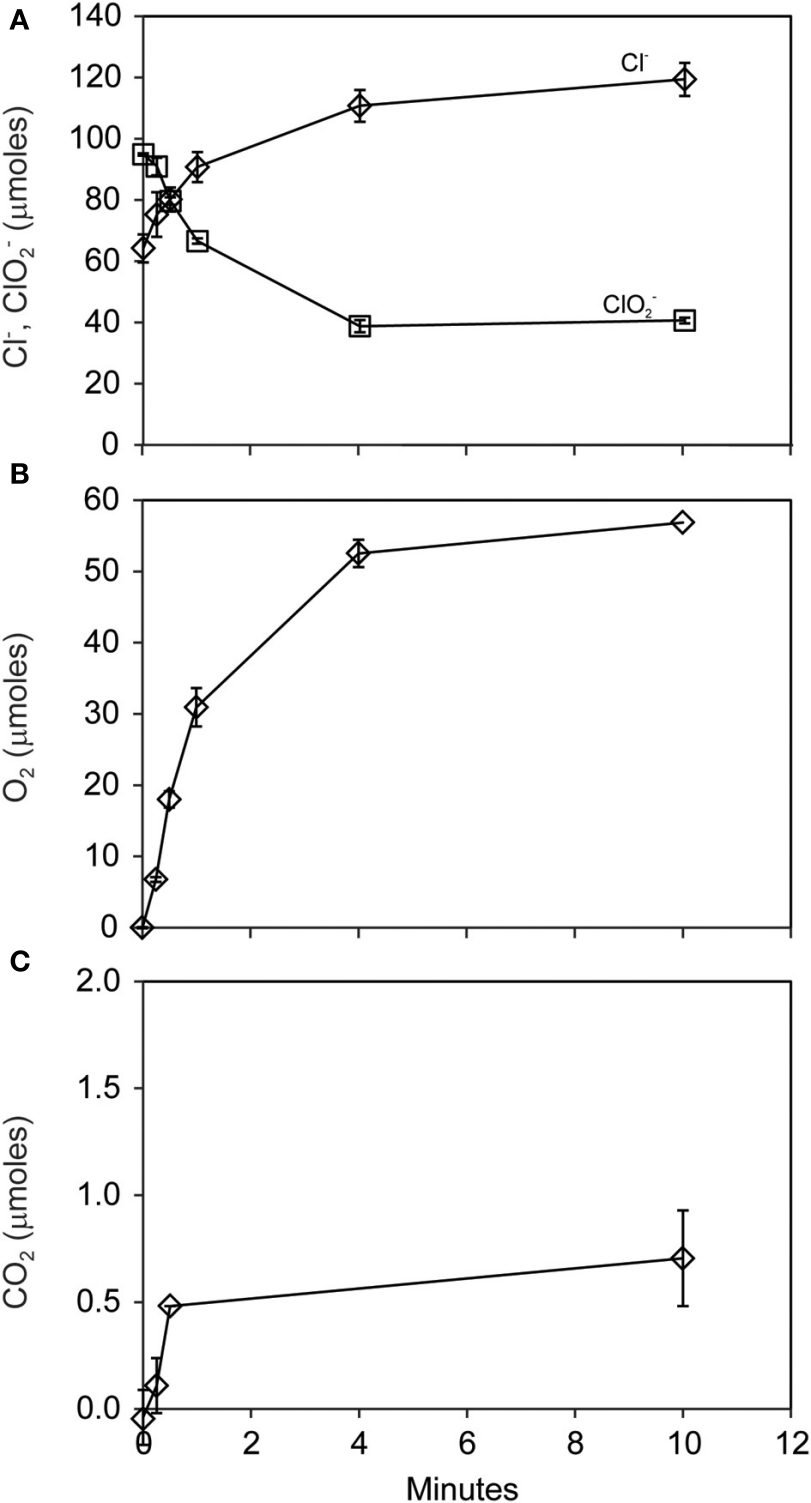
**Short-term course of reaction of *D. agitata* CKB following addition of 10 mM ClO^−^_2_, showing loss of ClO^−^_2_ (squares) and production of Cl^−^ (A) and production of O_2_ (B)**. A minor amount of CO_2_ was produced **(C)**. Symbols represent the mean and standard deviation of triplicate measurements. Absence of bars indicates that the error is smaller than the symbol size.

The pattern of early O_2_ production during ClO^−^_2_ dismutation was made clear in a subsequent short-term (10 min) experiment where 56 μmoles of both O_2_ and Cl^−^ were produced during the consumption of 56 μmoles of ClO^−^_2_ (Figures 3A,B). A minor amount of CO_2_ (<1 μmole) was produced (Figure 3C). Nearly half (40 μmoles) of the added ClO^−^_2_ remained unreacted at the end of the experiment.

### Mixed cultures oxidized CH_4_

**Figure 4:**
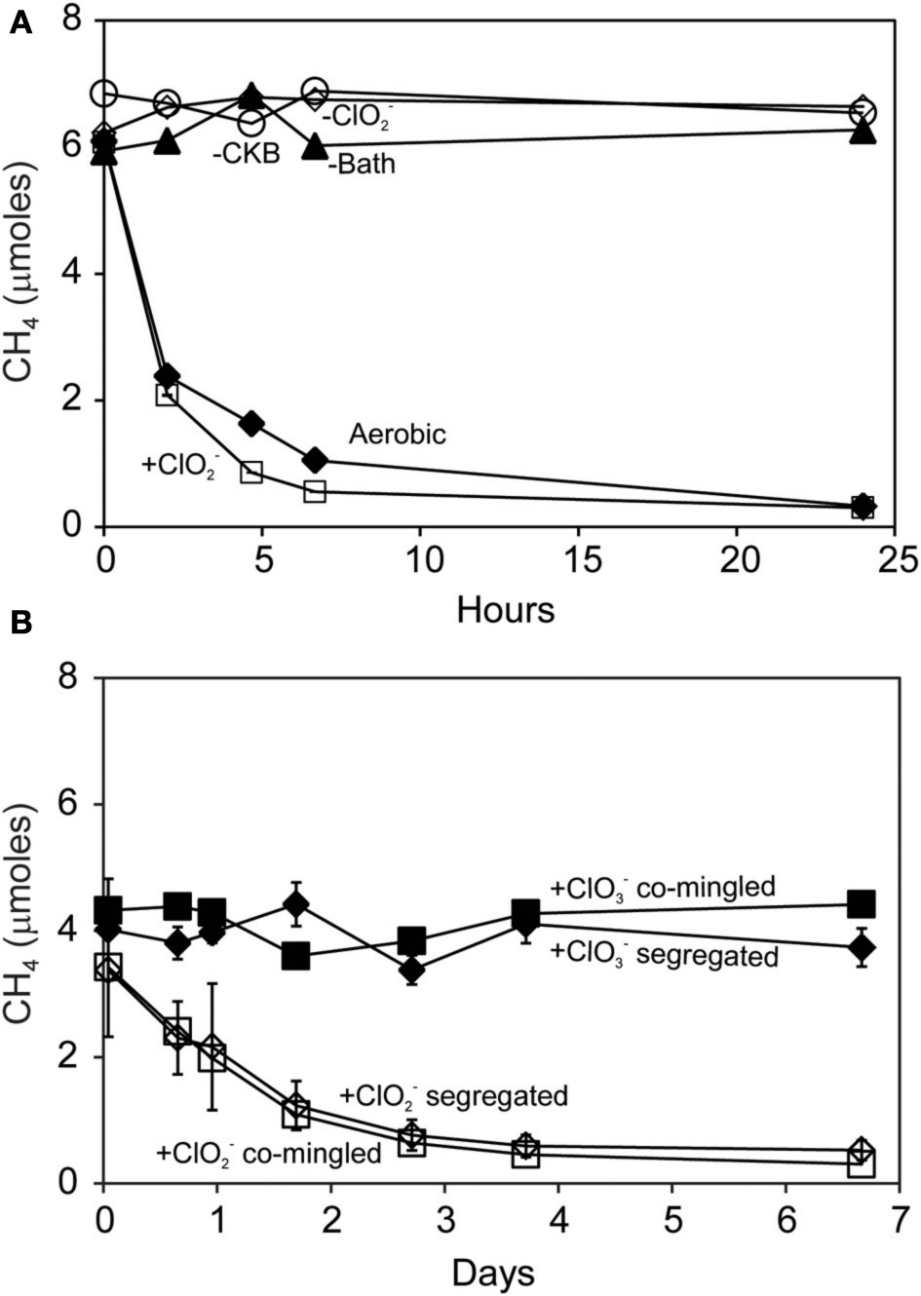
**Methane uptake by mixed cultures of *D. agitata* CKB with *M. capsulatus* Bath (A) and *M. album* BG8 (B) during anaerobic incubations**. Cultures in **(A)** were co-mingled and provided with 5 mM ClO^−^_2_ (open squares). Aerobic controls are also shown (solid diamonds). Negative controls were prepared by withholding *M. capsulatus* Bath (solid triangles), *D. agitata* CKB (open circles), or ClO_2−_ (open diamonds). Cultures in **(B)** were either co-mingled (squares) or segregated under a common headspace (diamonds) and provided with either ClO^−^_2_ (open symbols) or ClO^−^_3_ (solid symbols). Symbols represent the mean and standard deviation of triplicate measurements. Absence of bars indicates that the error is smaller than the symbol size.

Methane was oxidized by methanotrophic bacteria *M. capsulatus* Bath (Figure 4A) and *M. album* BG8 (Figure 4B) during the reaction of *D. agitata* CKB with ClO^−^_2_. No removal of CH_4_ was observed in mixed cell suspensions amended with ClO^−^_4_ or ClO^−^_3_ (i.e., no added ClO^−^_2_). Methane consumption occurred while the methanotrophs were in direct contact with up to 10 mM ClO^−^_2_. Methane removal during incubations of co-cultures of *D. agitata* CKB with *M. capsulatus* Bath at 37°C occurred within 1 day while removal of CH_4_ by *M. album* BG8 at 28°C occurred over 4 days. Similar rates of CH_4_ consumption were observed for these mixed cultures whether they were segregated or co-mingled (Figure 4B) indicating that direct contact of cells was not required for methane consumption to occur.

### Mixed cultures oxidize ^14^CH_4_ to ^14^CO_2_

**Figure 5:**
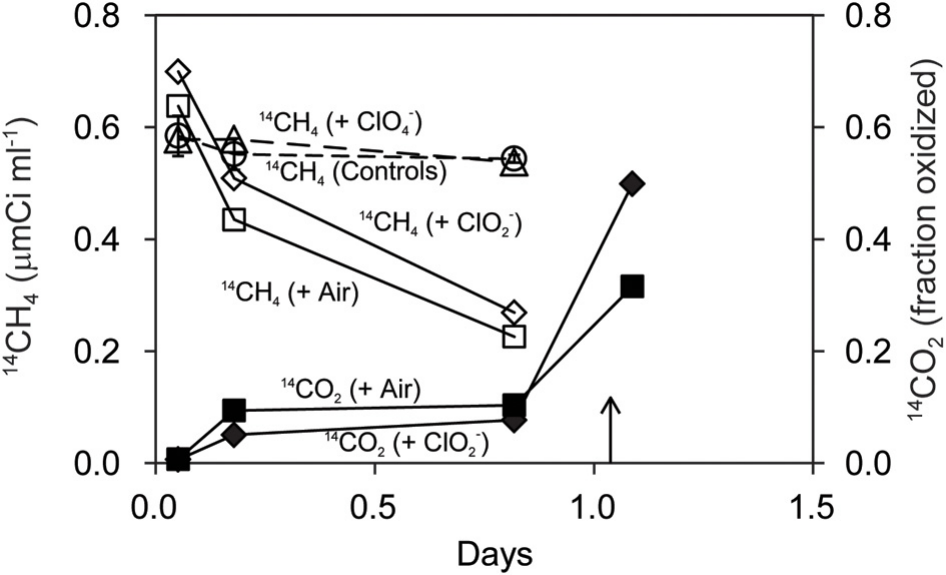
**Oxidation of ^14^CH_4_ (open symbols) to ^14^CO_2_ (closed symbols) by *M. trichosporium* OB3b during anaerobic incubations with *D. agitata* CKB following addition of ClO^−^_4_ (triangles) and ClO^−^_2_ (diamonds)**. Triangles represent the mean and standard deviation of triplicate measurements of samples with ClO^−^_4_ added. All others were single bottles. Controls (no bacteria or no chloroxyanion added) were pooled and are shown as circles with error bars. Aerobic incubations with *M. trichosporium* OB3b alone are also shown (squares). Samples were acidified at the time indicated by the arrow.

Strain *M. trichosporium* OB3b co-cultured with *D. agitata* CKB containing 5 mM acetate oxidized ^14^CH_4_ directly to ^14^CO_2_ (Figure 5). The rate of ^14^CH_4_ loss under anaerobic conditions with 5 mM added ClO^−^_2_ was similar to that under aerobic conditions. No loss of ^14^CH_4_ occurred in mixed cell suspensions amended with 5 mM ClO^−^_4_ in lieu of ClO^−^_2_ or in controls without methanotrophs. During the incubation, the product of methanotrophy (^14^CO_2_) was distributed about equally between liquid and gas phases. Roughly 20% of the added ^14^CH_4_ appeared as ^14^CO_2_ in the headspace after 20 h (0.8 days). Most of the ^14^CH_4_ added was recovered as ^14^CO_2_ (60–90% recovery after acidification).

### Oxidation of CH_4_by soils

**Figure 6:**
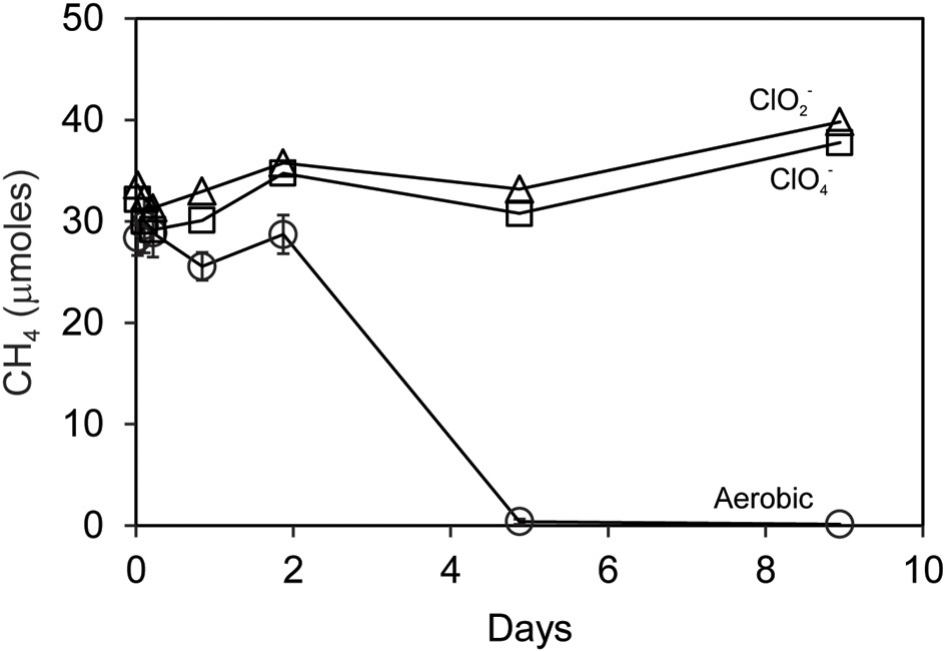
**Methane uptake by Searsville Lake sediment slurries pre-adapted to reduce perchlorate using acetate as electron donor**. Anaerobic incubations with 5 mM added ClO^−^_4_ (squares) or ClO^−^_2_ (triangles) showed no uptake. Aerobic incubations (circles) represent the mean and standard deviation of CH_4_ measurements in all bottles aerobic.

Searsville Lake sediment slurries removed repeated pulses of added ClO^−^_4_ during anaerobic incubations using freshwater media with added acetate. Over a period of 1 month, 4 additions of 10 mM ClO^−^_4_ were removed in bottles with commensurate consumption of 4 additions of 5 mM added acetate (data not shown). These slurries, thus enhanced in ClO^−^_4_ reducing capacity, were used in separate incubations with added CH_4_ (Figure 6). Under aerobic conditions, incubations with or without additions of ClO^−^_4_ or ClO^−^_2_ (5 mM) completely consumed 30 μmoles CH_4_ within 5 days. However, no oxidation of CH_4_ occurred during anaerobic incubations of Searsville Lake sediment slurries either with or without additions of ClO^−^_4_ or ClO^−^_2_.

**Figure 7:**
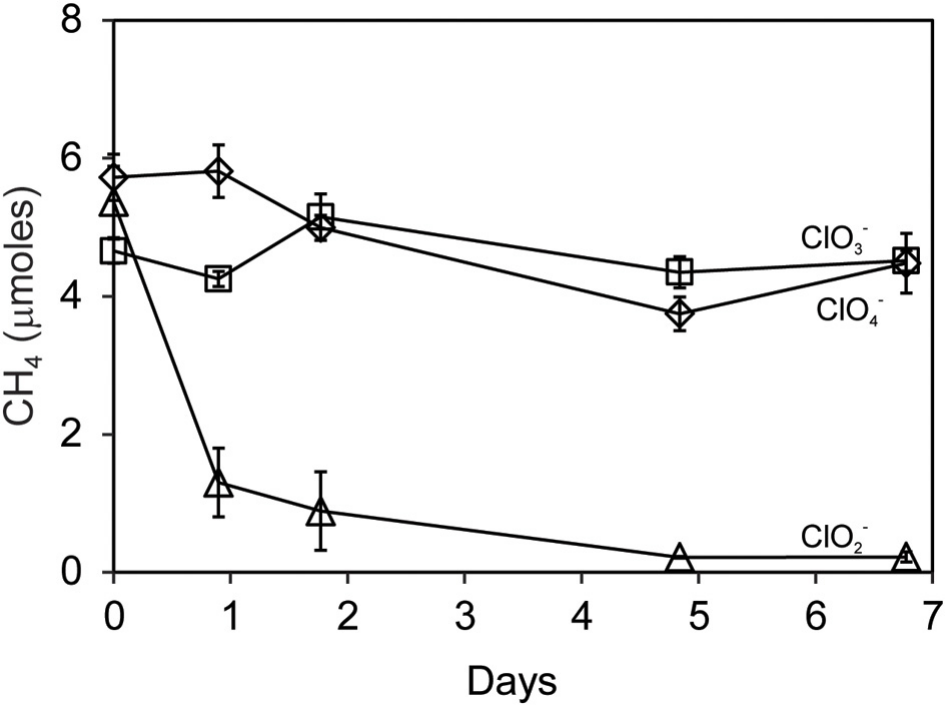
**Methane uptake by Searsville Lake soils during anaerobic incubations with *D. agitata* CKB following addition of ClO^−^_2_ (triangles), ClO^−^_4_ (diamonds), or ClO^−^_3_ (squares)**. Symbols represent the mean and standard deviation of triplicate measurements. Absence of bars indicates that the error is smaller than the symbol size.

Methane oxidation was observed when Searsville Lake soil (previously enhanced in methanotrophic activity) was segregated from liquid cultures of *D. agitata* CKB. Methane was completely consumed over the next 5 days by soil methanotrophs during the reaction of DPRB with 10 mM ClO^−^_2_ (Figure 7). No CH_4_ loss was observed when DPRB were provided with either 10 mM ClO^−^_4_ or ClO^−^_3_.

## Discussion

Few enzymes outside of photosystem II are capable of generating dioxygen in anoxic settings. In addition to chlorite dismutase (Cld), these include superoxide dismutase and catalase (McCord et al., 1971), and a putative nitric oxide dismutase (Ettwig et al., 2012). Chlorite dismutase has been purified from at least four DPRB (Mehboob et al., 2009) and is well studied (Coates et al., 1999b; Lee et al., 2008; Goblirsch et al., 2010, 2011). It is a heme enzyme which operates hyperselectively; its assumed sole function is to detoxify ClO^−^_2_. The present study is the first report of anaerobic methane oxidation linked to the combined presence of DPRB and ClO^−^_2_. We emphasize that this is a “cryptic” aerobic methane oxidation in that the organisms oxidizing methane are aerobic methanotrophs as opposed to anaerobic methanogenic archaea utilizing reverse methanogenesis to consume methane (Hinrichs et al., 1999). As such, this process is somewhat analogous to nitrite-dependent anaerobic methane oxidation as purportedly carried out by the mixed culture containing *Methylomirabalis oxyfera* (Ettwig et al., 2010). In our study, methanotrophs use O_2_ derived from disproportionation of ClO^−^_2_ by DPRB (reaction 1) to oxidize CH_4_ (reaction 3). This is in contrast to the mechanism proposed for *M. oxyfera* in which a single organism may use O_2_ from disproportionation of NO derived from NO_2_− (reaction 4) to oxidize CH_4_ (reaction 5):

(4) $$2\text{NO }\to \;{\text{N}}_{2}+{\text{O}}_{2}\;\Delta \text{G }{{}^{\circ}}^{\prime }=-173\text{ kJ/mol }{\text{O}}_{2}\;$$

(5) $$\begin{array}{l} 3{\text{CH}}_{4}+8{\text{NO}}_{2}^{-}+8{\text{H}}^{+}\;\to \;3{\text{CO}}_{2}+4{\text{N}}_{2}+10{\text{H}}_{2}O\\ \;\Delta \text{G }{{}^{\circ}}^{\prime }=-928\text{ kJ/mol }{\text{CH}}_{4} \end{array}$$

Previous work by Coates et al. (1998, 1999a); Coates and Achenbach (2006) demonstrated a link between Cld activity and other aerobic hydrocarbon oxidizing bacteria during the degradation of benzene and naphthalene. In their experiments with pristine soil and hydrocarbon contaminated sediment, ^14^C-benzene was oxidized to ^14^CO_2_ over several days when provided with ClO^−^_2_ in the presence of washed cells of *D. agitata* CKB under anoxic conditions. Similarly, ^14^C-napthalene was rapidly oxidized to ^14^CO_2_ in the presence of washed cells of *D. agitata* CKB and *Pseudomonas* sp. strain JS150 (an aerobic hydrocarbon oxidizer) when provided with ClO^−^_2_.

Here we demonstrated that O_2_ released by the reaction of ClO^−^_2_ with pure cultures of DPRB could be utilized by a variety of methane oxidizing bacteria, including γ-Proteobacteria (*M. capsulatus* Bath and *M. album* BG8) and α-Proteobacteria (*M. trichosporium* OB3b) methanotrophs. Addition of 10 mM ClO^−^_2_ to DPRB resulted in only 40–60% recovery as O_2_ and Cl^−^ (Figure 3). This may be attributed to a toxic effect of elevated ClO^−^_2_ or bleaching of the Cld enzyme (Streit and DuBois, 2008). Nonetheless, much of the available O_2_ was freely released during the reaction of ClO^−^_2_ with *D. agitata* CKB, consistent with localization of Cld in the periplasm of DPRB (O'Connor and Coates, 2002). We demonstrated that direct addition of 5 or 10 mM ClO^−^_2_ to mixed cultures of DPRB and methanotrophs did not inhibit the methanotrophs. We also showed that direct contact between the cells was not required as CH_4_ oxidation also occurred when the cells were contained in separate compartments under a common headspace. We further showed that ^14^CH_4_ was quantitatively oxidized to ^14^CO_2_ by the methanotrophs in culture. Our conclusion is that methane oxidizers utilized O_2_ provided by the dismutation of ClO^−^_2_ by DPRB.

We were unable to link methane oxidation to perchlorate or chlorate reduction. Small amounts of oxygen were produced when cultures of *D. agitata* CKB were amended with ClO^−^_4_ or ClO^−^_3_ (Figure 2), however methane was not consumed during co-culturing with methanotrophs (Figures 4, 6). This was previously observed for benzene and naphthalene (Coates et al., 1999b; Coates and Achenbach, 2006) and may be explained by O_2_ scavenging attributable to other processes, including activation of a terminal oxidase during (per)chlorate reduction (Rikken et al., 1996). It is also possible that slower kinetics of ClO^−^_4_ and ClO^−^_3_ reduction limits the production and subsequent dismutation of ClO^−^_2_ and therefore release of O_2_. Relief of this bottleneck could lead to more O_2_ being available to aerobic methanotrophs and stimulation of the unique process described herein.

*In-situ* oxidation of CH_4_ using O_2_ derived from chlorite dismutation may be useful in removing elevated levels of CH_4_ in subsurface environments. Chlorite is 10^4^ times more soluble in water than O_2_ and could be easily and safely directed to the anaerobic zone (where methane may be present) during bioremediation. One example is enhanced oxidation of landfill methane without the use of forced air, reducing the risk of fire and explosion. The ability of methanotrophs and DPRB to function in separate compartments under a common headspace could be exploited at distal stages of oil development, for instance at well heads where unusable CH_4_ is typically flared off to reduce transportation costs or risk. In addition, production of CO_2_ formed during oxidation of CH_4_ may be viewed similarly to injected CO_2_ in efforts to dissolve and flush oil from developed petroleum reservoirs (Blunt et al., 1993). Further, bioclogging by cells and biocementation resulting from carbonate precipitation may be enhanced by the growth and activity of microbes capable of linking methane oxidation with (per)chlorate reduction. Bioclogging and biocementation are features of microbial enhanced hydrocarbon recovery most likely to be exploited by geotechnologists to direct hydrocarbon flow into more permeable substrates in order to enhance recovery. However, it must be noted that we observed no methane oxidation in water saturated soils exposed to both CH_4_ and ClO^−^_2_ (Figure 6) indicating that O_2_ derived from chlorite dismutation may face transport limitations in saturated anaerobic environments and may be consumed before reaching nearby CH_4_. Even under our unsaturated experimental conditions where liquid cultures and soil were segregated under a common headspace (Figure 7) the amount of soil present in each microcosm had to be optimized in order to balance methanotrophy with other soil O_2_ utilizing processes.

Oxidation of CH_4_ by the mechanism identified here may reduce the greenhouse impact of fugitive gases during hydrocarbon reservoir development and recovery because the global warming potential of CO_2_ is 25 times lower than CH_4_ on a 100 year time scale (IPCC 5th assessment, 2013). In addition, intermediates along the pathway of aerobic methane oxidation (e.g., methanol, formaldehyde, and formate) are themselves quite useful as chemical feedstocks for a myriad of industrial applications including biofuel production.

### Conflict of interest statement

The Guest Associate Editor Hans Carlson declares that, despite having collaborated with author John Coates, the review process was handled objectively. The authors declare that the research was conducted in the absence of any commercial or financial relationships that could be construed as a potential conflict of interest.
